# PI3K/ c-Myc/AFF4 axis promotes pancreatic tumorigenesis through fueling nucleotide metabolism

**DOI:** 10.7150/ijbs.77150

**Published:** 2023-03-27

**Authors:** Chenming Ni, Wenyu Liu, Kailian Zheng, Shiwei Guo, Bin Song, Wei Jing, Gang Li, Bo Li, Canrong Ni, Keqing Shi, Gang Jin, Guanzhen Yu

**Affiliations:** 1 Department of Pancreatic Surgery, Changhai Hospital, Naval Medical University, Shanghai, 200433, China.; 2 Department of Pathology, Changhai Hospital, Naval Medical University, Shanghai, 200433, China.; 3 Precision Medical Center Laboratory, The First Affiliated Hospital of Wenzhou Medical University, Zhejiang Province, 325000, China.

**Keywords:** AFF4, Pancreatic cancer, PI3K, c-Myc, Nucleotide metabolism

## Abstract

MLL-AFF4 fusion gene has been discovered in acute leukemia, whether AFF4 alone plays a role in tumor, especially pancreatic tumorigenesis, is still elusive. Increasing evidence suggests that cancer cells altered nucleotide metabolism during tumorigenesis. In present study, we observed AFF4 overexpression promoted cell proliferation, colony formation and cell cycle progression while loss of AFF4 impairs above phenotypes of pancreatic ductal carcinoma (PDAC) cells. Using RNA-profiling, we revealed that HPRT1 and IMPDH2, two enzymes in the nucleotide metabolism pathway, were upregulated following AFF4 overexpression. Simultaneous expression of HPRT1 and IMPDH2 would mainly rescue the phenotypes of cells lacking AFF4. Additionally, xenograft study proved HPRT1 and IMPDH2 genetically function in the downstream of AFF4, which was recruited by PAX2 when CDK9 mediated AFF4 phosphorylation at S388 and drove HPRT1 and IMPDH2 expression. We further discovered PI3K/c-Myc axis is required for AFF4 expression in PDAC cells. Finally, we obtained the positive correlation between c-Myc and AFF4 or AFF4 and HPRT1/IMPDH2 in clinical PDAC samples. Otherwise, we conducted data-mining and found that the expression levels of AFF4 and HPRT1/IMPDH2 are correlated with patients' prognosis, establishing AFF4 as a potential biomarker and therapeutic target for PDAC.

## Introduction

AFF4 is a core subunit of super elongation complex (SEC), which is required for efficient transcription through release of RNA polymerase II (Pol II) with its positive transcription elongation factor b (P-TEFb) and promoting transcriptional sustainability with ELL2 subunit in SEC. Recently, the structure of P-TEFb/AFF4/ELL2 complex has been resolved with high resolution. Given this complex initiates HIV gene transcription 1-4, the discovery of inhibitors aiming to disrupt the interaction between AFF4 and P-TEFb is the research hotspot of HIV. For example, Liang et al. identified peptidomimetic lead compounds, KL-1 and its structural homolog KL-2, which disrupt the interaction between the SEC scaffold protein AFF4 and P-TEFb, resulting in an impaired release of RNA Pol II from promoter-proximal pause sites and a reduced rate of constitutive transcription elongation 5.

As the special ability of prompting expression, malfunction of SEC contributes to multiple human diseases, including cancers, thus AFF4 was also involved in cancer progression. Such as, AFF4 upregulates SOX, which then promotes tumorigenesis and tumor-initiation capacity of head and neck squamous cells 6. AFF4 also facilitates melanoma progression by upregulating c-Jun transcriptional activity 7. Dysregulation of AFF4 expression leads to tumorigenesis 8, 9. Together, robust expression of AFF4 sustains multiple kinds of cancer progression, however, the role of AFF4 in pancreatic cancer remains unclear.

Nucleotide metabolism, including purine and pyrimidine metabolism, is inevitably linked to cell cycle progression and offers the therapeutic target for dealing with PDAC. The oncogenic KRAS mutation has a critical role in the initiation of human PDAC by altered nucleotide synthesis 10. In this study, we found two genes *HPRT1* and *IMPDH2* were downregulated upon depletion of *AFF4*. Both of HPRT1 and IMPDH2 execute their enzymatic activity in the guanine nucleotide synthesis pathway. However, we observed the inhibition of phosphatidylinositol-3 kinase (PI3K) activity, but not KRAS, reduced AFF4 expression, and in turn downregulated HPRT1 and IMPDH2.

PI3Ks are lipid kinases that convert signaling lipid PIP2 to PIP3. PIP3 molecules recruit proteins bearing PIP3-binding pleckstrin homology (PH) domains, such as AKT to the plasma membrane 11. In the past decades, PI3K signaling pathway is believed to be deregulated in a wide range of human cancers. Except AKT, PI3K also communicates with other signaling pathways, such as MAPK cascades 12, 13. In this study, we observed a downregulation of AFF4 mRNA accompanying inhibition of PI3K, ERK or AKT. Generally, PI3K acts in the upstream of AKT or ERK. Hence, in this study, PI3K could transduce signals to AKT and have a crosstalk with ERK to sustain AFF4 expression in PDAC cells.

The metabolic reprogramming required for cell proliferation and survival is mainly mediated by c-Myc in most of tumor cells. In this study, c-Myc directly drives *AFF4* expression, which is required for prompting HRPT1 and IMPDH2 expression. Thus, we propose that PI3K/c-Myc mediated AFF4 expression drives cell cycle progression through boosting HPRT1 and IMPDH2 expression in PDAC cells. Given the oncogenic role of AFF4 has been well-explored, our study offers a therapeutic target for pancreatic cancer progression.

## Materials and Methods

### Patient specimens

Tumors and paired normal pancreatic tissues were obtained from patients with PDAC who underwent surgery at Changhai Hospital, Naval Medical University, Shanghai. All patients signed the informed consent for basic research in this study. This study protocol was approved by the ethics committee of Changhai Hospital. Written informed consent was obtained from all participants in this study. All research was performed in accordance with the provisions of the Declaration of Helsinki of 1975.

### Plasmids

pCDH-AFF4, pCDH-HPRT1 (Puromycin) or pCDH-IMPDH2 (Hygromycin) was constructed by cloning the open reading frame (ORF) of AFF4, HPRT1 or IMPDH2 with template cDNA from BxPC-3 cells.

The pGIPZ control or pGIPZ AFF4 shRNA was generated by inserting a small hairpin RNA targeting GFP or human AFF4 ORF (NM_014423.4) with a sequence 5'-cccaagcctacagtaccacca-3' for #1 or 5'- cctccatctgcaccaactctt-3' for #2 into the pGIPZ vector.

### Liquid chromatography-based mass spectrum (LC-MS) for guanine determination

After the cells were cleaved by 80% methanol, the lysates were collected by high-speed centrifugation. The fraction was performed on a C18 column with 1% acetic acid: acetonitrile: methanol (90:8:2) as mobile phase in 25^o^C, and flow rate was 1 ml/min. Data were collected for LC-MS/MS analysis, which was carried out with an EASY-nLC 1000 liquid chromatograph (Thermo Fisher Scientific) coupled to an Orbitrap Fusion mass spectrometer (Thermo Fisher Scientific).

### Subcutaneous xenograft model

In brief, 2 × 10^6^ BxPC-3 stable cells (Figure 4) were subcutaneously injected into the left dorsal flank of randomized 6-week-old female athymic nude mice. After inoculation for 23 days, mice were euthanized, and tumors were dissected. Differences in weight, growth rate, and indicated protein levels of xenograft tumors were statistically analyzed.

### IHC analysis

Tissue sections from paraffin-embedded human PDAC tissues were stained with antibodies as indicated. We quantitatively scored tissue sections according to the percentage of positive cells and staining intensity. We rated the intensity of staining on a scale of 0-3: 0, negative; 1, weak; 2, moderate; and 3, strong. We assigned the following proportion scores: X indicates the percentage of tumor cells that were stained (0 ≤ [X1 + X2 + X3] ≤ 100), where X3 indicates strong staining, X2 moderate staining and X1 weak staining. The score (H-score) was obtained using the following formula: 3 × X1 + 2 × X2 + 1 × X3, giving a range from 0 to 300.

### Statistical analysis

All results are presented as the mean ± standard error of the mean (SEM) or SD unless stated otherwise. Statistical analysis was performed using GraphPad Prism Version 7.04 (GraphPad Software, Inc.). A p-value of <0.05 was considered significant. *p < 0.05, **p < 0.01, ***p < 0.001. Pearson's correlation analyses were used to calculate the regression and correlation between two groups. Log-rank test was used to measure patient survival.

## Results

### AFF4 is required for in pancreatic cancer cell proliferation, colony formation and cell cycle progression

**Figure 1:**
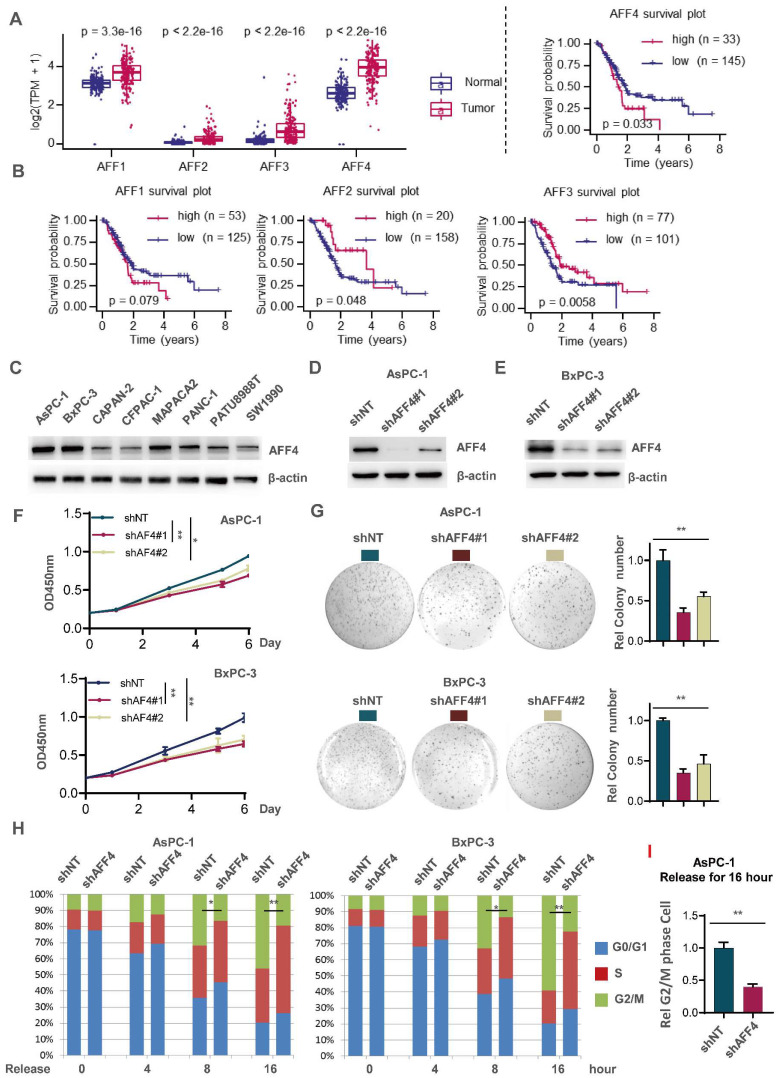
** AFF4 depletion restrains pancreatic cancer cell proliferation with a delayed cell cycle.** (A-B) AFF1-4 mRNA expression levels (TPM) in PDAC tumor and normal tissues (A) and survival curves of AFF1-4 with best cutoff separation using expression levels (B) were mined through human TCGA-PDAC database. (C-E) Western blot analysis of AFF4 protein levels in eight PDAC cell lines or followed cell line constructions was performed. (F-H) Cell proliferation assay (F), colony formation assay (G) and cell cycle assay (H) were performed in AsPC-1 or BxPC-3 shNT or shAFF4#1, #2 cells. (I) AsPC-1 cells with or without AFF4 were released for 16 hrs from cell cycle arrest, then collected for PI staining and cell cycle analysis. NT, non-target. Data in triplicate from (G and I). F, two-way ANOVA. G-I, two tailed Student's t-test. (*p < 0.05; **p < 0.01).

To ensure the significance of our study, we first extracted mRNA expression of AFF1-4 genes in pancreatic normal and tumor tissues through datamining human TCGA-PDAC database. As shown in Figure 1A, the expression of AFF1-4 in PDAC tissues consistently displayed a higher level than those in normal tissues, suggesting the members of AF4/FMR2 family would exert the progressive function for pancreatic cancer (Figure 1A). To further explore the prognostic significance of AFF1-4 in PDAC, the survival curves of AFF1-4 were also plotted by mining TCGA-PAAD database and showed that higher levels of both AFF1 and AFF4, especially AFF4 mRNA, predict worse prognosis in PDAC patients, while the overall survival curves of AFF2/3 indicated that low expression of AFF2/3 conveys worse prognosis (Figure 1B). Because the better consistence of AFF4 expression in tumor and normal with survive plot existed in PDAC, we next focused on AFF4 and detected AFF4 protein levels in eight PDAC cell lines, and among these cell lines, AsPC-1 and BxPC-3 expressed higher levels of AFF4 than other cell lines, while CAPAN-2, CFPAC-1 and SW1990 expressed the lowest protein levels of AFF4 (Figure 1C). We then successfully knocked down *AFF4* in AsPC-1 or BxPC-3 using two independent small hairpin RNA (shRNA) pairs individually targeting AFF4 mRNA (Figure 1D and 1E). AFF4 depletion impaired cell proliferation measured by CCK8 (Figure 1F) and inhibited the capacity of anti-anoikis measured by colony formation (Figure 1G), which proved AFF4 could play as an oncogenic role in PDAC. Among two independent shRNA pairs individually targeting AFF4 mRNA, the shAFF4#1 targeting sequence showed a better knockdown effect in PDAC cells and consistently obtained a more severe damage, in terms of proliferation and anti-apoptosis. In line with above data from cell proliferation and colony formation assays, a cell cycle study showed the cells lacking AFF4 presents a delayed cell cycle progression in G2/M phase and probably caused the proliferation arrest and sensitivity to anti-anoikis (Figure 1H).

### Overexpression of AFF4 promotes PDAC cell proliferation and an accelerated cell cycle

Next, we overexpressed AFF4 in both CAPAN-2 and CFPAC-1 cells (Figure S1A). AFF4 overexpression moderately promoted cell proliferation measured by CCK8 (Figure S1B) and acquires the capacity of anti-anoikis measured by colony formation (Figure S1C). How the elevated expression of AFF4 works in PDAC cells is still unclear. A cell cycle study showed that the cells with AFF4 overexpression presents a more aggressive cell cycle progression as more cells in the overexpression group enter the G2/M phase than control cells (Figure S1D), which partly explains that PDAC cells with AFF4 overexpression have a faster cell proliferation and a stronger resistance to anoikis. Taken together, as a core subunit of SEC, AFF4 is required for cell cycle progression and anti-anoikis. We repeated this cell cycle analysis using AsPC-1 and CAPAN-2 cells, confirmed that AFF4 is required for PDAC cells crossing G2/M phase (Figure 1I and S1E). Finally, 5-ethynyl-2'-deoxyuridine (EdU) staining assay showed AFF4 mediated cell cycle progression is required for cell proliferation (Figure S1F-G). Hence, we preliminarily established an oncogenic role of AFF4 through driving cell cycle progression of PDAC cells.

### AFF4 is required for the expression of HPRT1 and IMPDH2

**Figure 2:**
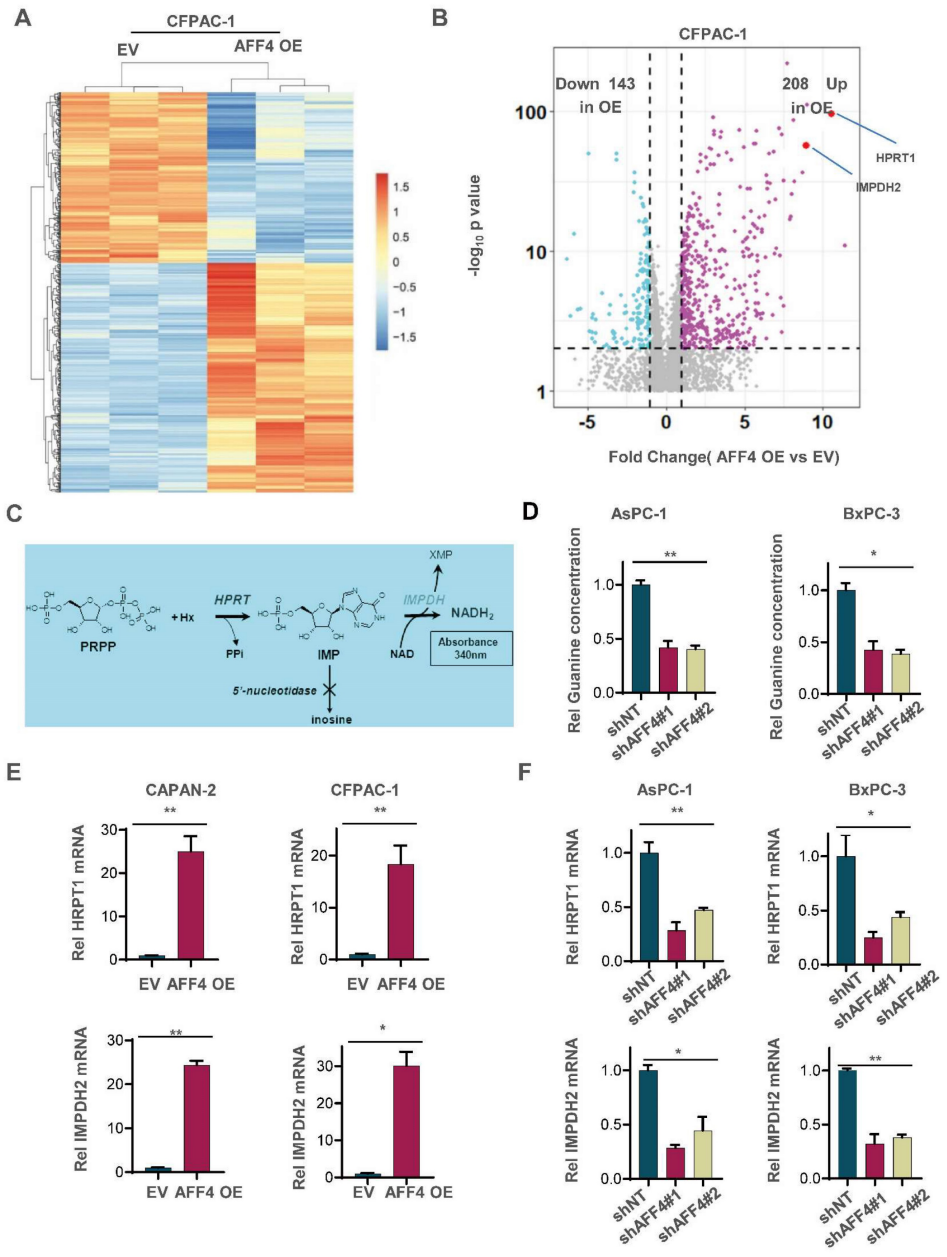
** AFF4 is required for the expression of HPRT1 and IMPDH2.** (A-B) RNA-seq analyses were performed in CFPAC-1 cells stably expressing EV or AFF4 OE. The differential genes with fold change >2 and p value<0.05 was presented (A). Differentially expressed genes, *HPRT1* and *IMPDH2* are marked (B). (C) The catalyzed reaction of HPRT1 and IMPDH2 in the nucleotide metabolic pathways. (D) In AsPC-1 and BxPC-3 cells with or without AFF4, relative concentration of guanine is determined using LC-MS. (E-F) The mRNA levels of HPRT1 and IMPDH2 were determined by qPCR using specific primers against HPRT1 or IMPDH2 mRNA in CAPAN-2 and CFPAC-1 cells (E) or AsPC-1 and BxPC-3 cells (F) with genetic manipulation. Data in triplicate from (D-F). D-F, two tailed Student's t-test. (*p < 0.05; **p < 0.01).

To uncover how AFF4 drives cell cycle progression, we performed the RNA profiling to reveal AFF4 mediated gene-expression. A heat map showed that the changed gene-expression of RNA profiling in CFPAC-1 cells with or without AFF4 overexpression. 208 genes were significantly up-regulated, and 143 genes were significantly down-regulated following AFF4 overexpression (Figure 2A and 2B). Among the 208 up-regulated genes, *HPRT1* and *IMPDH2* are two genes encoding enzymes in the nucleotide metabolic pathways, especially IMPDH2 as the rate limited enzyme in during guanine biosynthesis, measured by the intracellular level of inosine (Figure 2C). Then, we measured the levels of intracellular guanine and found the loss of AFF4 showed a significant decrease of guanine in PDAC cells (Figure 2D) while overexpression of AFF4 significantly upregulated the intracellular guanine and IMPDH2 produced metabolite hypoxanthine monophosphate (XMP) (Figure S2A and S2B). We also tested the expression levels of HPRT1 and IMPDH2 in CAPAN-2 or CFPAC-1 cells accompanying expressing vehicle (EV) or AFF4 overexpression (AFF4 OE). As shown when AFF4 was overexpressed, the expression levels of HPRT1 and IMPDH2 were also increased by 15-30 times (Figure 2E). While AFF4 was depleted in AsPC-1 or BxPC-3 cells, the expression levels of HPRT1 and IMPDH2 were decreased by 50-75% (Figure 2F). Thus, as AFF4 is a core subunit of SEC, *HPRT1* and *IMPDH2* could be the two genetic downstream targets of AFF4.

### Simultaneous expression of HPRT1 and IMPDH2 in PDAC cells lacking AFF4 restores tumor cell function

**Figure 3:**
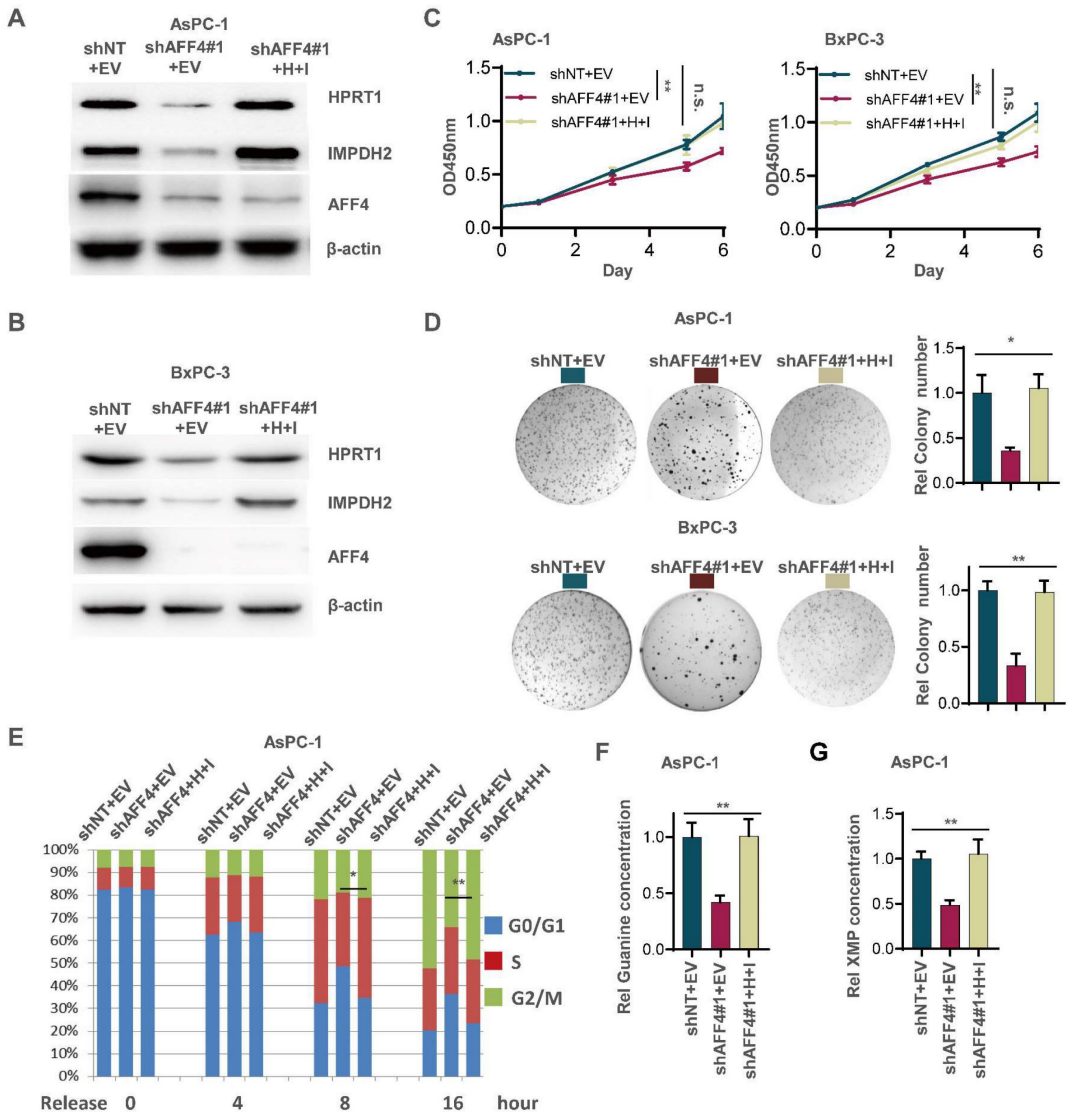
** Simultaneous expression of HPRT1 and IMPDH2 in PDAC cells lacking AFF4 restores cell proliferation, colony formation and cell cycle progression.** (A-B) Western blot confirmed the successful construction of ASPC-1 or BxPC-3 cell lines stably depleting AFF4 with restored simultaneous expression of HPRT and IMPDH2. (C-E) Cell proliferation assays (C), colony formation assays (D) and cell cycle assays (E) were performed in AsPC-1 or BxPC-3 cells expressing shAFF4 with or without restored simultaneous expression of HPRT1 and IMPDH2. (F-G) In above cells constructed in (A), relative intracellular concentration of guanine (F) or XMP (G) is determined by using LC-MS. H, HPRT1; I, IMPDH2. Data in triplicate from (D and F-G). C, two-way ANOVA. D-G, two tailed Student's t-test. (n.s., not significant; *p < 0.05; **p < 0.01).

Although the expression level of HPRT1 and IMPDH2 was changed accompanying AFF4 overexpression or depletion, we need to clearly identify the regulatory circuit of AFF4/HPRT1 and AFF4/IMPDH2. Thus, we simultaneously forced the expression of HPRT1 and IMPDH2 in the cells lacking AFF4. Western blot showed the successful construction of such stable cell lines used AsPC-1 or BxPC-3 as parent cells (Figure 3A and 3B). As shown in Figure 3C-3E, the restored expression of HPRT1 and IMPDH2 mainly rescued the functional defects of tumor cells lacking AFF4, such as cell proliferation (Figure 3C), colony formation (Figure 3D) and cell cycle progression (Figure 3E) to varying degrees, robustly indicating HPRT1 and IMPDH2 are two major downstream targets of AFF4 and the function of AFF4 in PDAC cells mainly depends on driving the expression of *HPRT1* and *IMPDH2* genes. Further, we also tested the relative guanine concentration in the above cells and confirmed that AFF4 depletion reduced the intracellular guanine biosynthesis and the supplemental expression of both HPRT1 and IMPDH2 would restore the relative guanine and XMP in the cells lacking AFF4 (Figure 3F and 3G, S3A and S3B), proving AFF4 mediated expression of HPRT1 and IMPDH2 would sustain nucleotide metabolism.

To further identify the regulatory circuit of AFF4 with HPRT1 or IMPDH2, and given IMPDH2 is the rate limiting enzyme in the guanine synthesis, we specifically inhibited IMPDH2 using the inhibitor mycophenolic acid (MPA) in the cells overexpressing AFF4. As shown in Figure S3, the treatment of PDAC cells with MPA, we observed a dramatic decrease or reduction of cell proliferation (Figure S3C), colony formation (Figure S3D) and cell cycle progression (Figure S3E) in PDAC cells overexpressing AFF4, robustly proving IMPDH2 functions in the downstream of AFF4.

### Simultaneous expression of HPRT1 and IMPDH2 restores solid tumor formation in PDAC cells lacking AFF4

**Figure 4:**
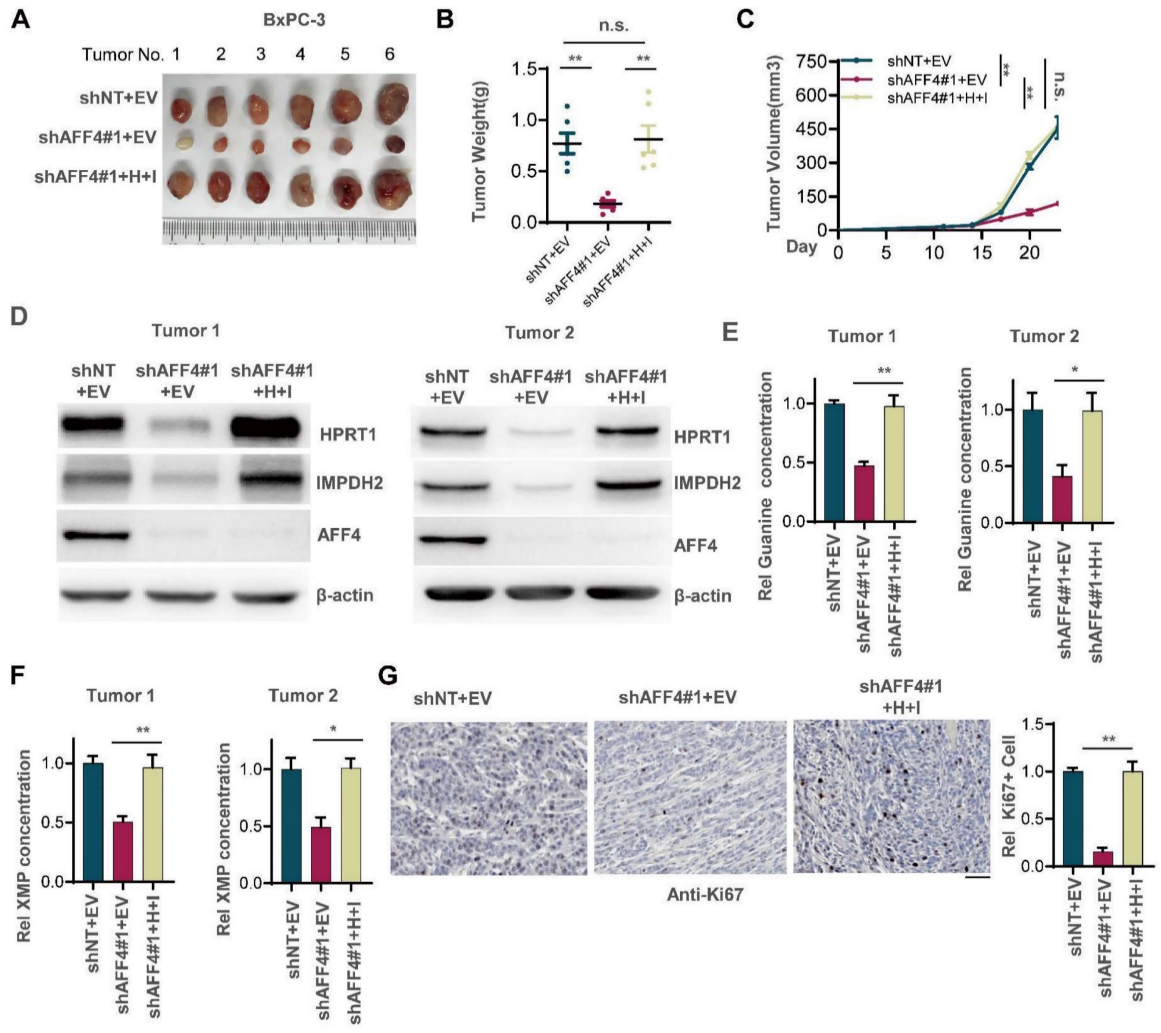
** Simultaneous expression of HPRT1 and IMPDH2 in PDAC cells lacking AFF4 restores solid tumor formation.** (A-C) The stable cell lines constructed in Figure 3B were transplanted into the left groin of nude mice (n=6, each group). Solid tumors were dissected once the largest tumor achieved 1 cm^3^ and imaged at the end of the experiment (A). Tumor weight was measured (B), and tumor volume was recorded every three days (C). (D) Western blot analysis of AFF4, HPRT1 and IMPDH2 expression in solid tumors derived from cells in Figure 3. (E-F) In solid tumors formed by cells constructed in Figure 3B, relative concentration of guanine (E) or XMP (F) is determined using LC-MS. (G) Ki67 staining was performed to examine proliferating cells in solid tumors. Scale bar, 100 μm. Data in triplicate from (E-G). B and E-G, two-tailed Student's t-test. C, two-way ANOVA. (n.s., not significant; *p < 0.05; **p < 0.01).

To examine whether loss of AFF4 exerts disadvantages on the formation of solid tumors and simultaneous expression of HPRT1 and IMPDH2 restores solid tumor formation, we transplanted BxPC-3 cells with indicated genetic manipulation into the left groin of nude mice. After 23 days, we excised tumors from nude mice and imaged solid tumors. As shown in Figure 4A-C, AFF4 depletion impaired tumor formation, and rescued simultaneous expression of HPRT1 and IMPDH2 recovered tumor formation and tumor growth (Figure 4A-C). We randomly tested the protein levels of AFF4 and HPRT1, IMPDH2 in two groups of xenograft tumors and obtained consistent results with cell lines, proving that the solid tumors were derived from BxPC-3 cells in Figure 3B (Figure 4D). We also tested the relative guanine concentration in the above solid tumors and observed the same result in Figure 3F that showed a decreased guanine and XMP in AFF4 deficient cells (Figure 4E and 4F). Furthermore, IHC was performed using an antibody against Ki67 and we observed that simultaneous expression of HPRT1 and IMPDH2 promoted tumor growth from cells lacking AFF4, proving a genetic axis of AFF4/HPRT1& IMPDH2 is existed in PDAC cells (Figure 4G).

### AFF4 drives *HPRT1* and *IMPDH2* expression depending on transcriptional factor PAX2

**Figure 5:**
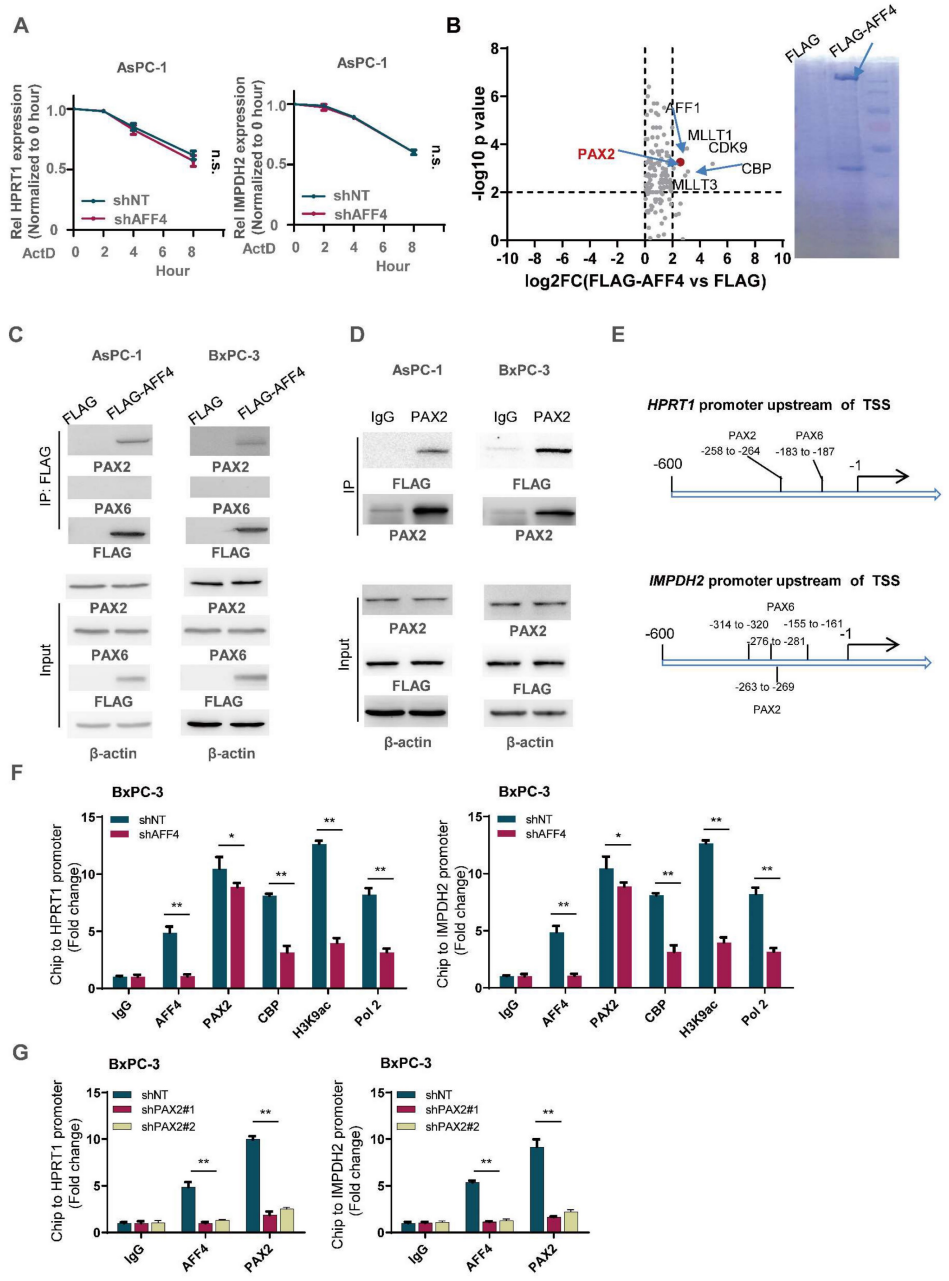
** AFF4 drives the genes expression of HPRT1 and IMPDH2 depending on transcriptional factor PAX2.** (A) mRNA decay assay. AsPC-1 cells expressing shNT or shAFF4 were incubated with ActD for indicated times. act D, 1μM. Primers against HPRT1 or IMPDH2 were used to perform qPCR. (B) FLAG-AFF4 was immunoprecipitated from BxPC-3cells, while the pCDH empty vector was used as a blank control. FLAG-AFF4 associated proteins were identified by LC-MS/MS. Fold change was evaluated by the ratio of FLAG-AFF4 vs FLAG. The x-axis represents log2 transformed fold change, and the y-axis represents minus log10 transformed p-values. A potential interactor with -log_10_ p value > 4 and log_2_ (fold change) > 2 was regarded as a potentially significant protein associated with AFF4. The enlarged red point indicates the only TF, PAX2. (C) Schematic diagram of predicted TF binding by PAX family, PAX2 and PAX6 on the promoter of HPRT1 and IMPDH2. (D) FLAG-AFF4 was transiently transfected into AsPC-1 or BxPC-3 cells, and FLAG-M2 beads were used to immuno-precipitate FLAG-tagged AFF4. (E) Reverse immunoprecipitation was performed using an antibody against PAX2. (F) ChIP assay was performed using antibodies against AFF4, PAX2, CBP, H3K9ac and RNA pol Ⅱ in BxPC-3 cells expressing shNT or shAFF4. (G) ChIP assay was performed using antibodies against AFF4 and PAX2 in BxPC-3 cells expressing shNT or shPAX2. Data in triplicate from (F-G). A, two-way ANOVA. F-G, two-tailed Student's t-test. (n.s., not significant; *p < 0.05; **p < 0.01).

To exclude the possibility of AFF4 mediating the mRNA stability of *HPRT1* and *IMPDH2* genes, we tested the mRNA decay using Actinomycin D, which is an inhibitor to prevent mRNA chain elongation through binding DNA. We observed the same trend of mRNA decay half-time in the cells expressing shNT or shAFF4, meaning AFF4 is not required for maintain the mRNA stability of *HPRT1* and *IMPDH2* (Figure 5A). As AFF4 is a subunit of SEC, it targets the gene promoter mainly depending on transcriptional factors.

To identify the transcription factor association with AFF4, we transfected FLAG-tagged AFF4 or FLAG control plasmid into PANC-1 cells and performed an immunoprecipitation (IP) assay. A FLAG-tagged AFF4 associated complex was identified by LC-MS/MS, and analysis of the differentiated proteins by a ratio of FLAG-AFF4 group versus FLAG control group is presented as a volcano map, revealing only one transcriptional factor, PAX2. The other proteins significantly associated with AFF4, such as MLLT1/3 and CDK9, have been also shown in BioGrid (Figure 5B). As AFF4 simultaneously drives *HPRT1* and *IMPDH2* expression, we suspect there could be PAX transcriptional factor binding site on the promoter of *HPRT1* and *IMPDH2* genes. Hence, we predicted the PAX binding site on the promoters of these two genes (Figure 5C). Next, we examined the interaction between AFF4 and PAX2/6, and the association of AFF4 with PAX2 was proven by forward and reverse Co-IP (Figure 5D and 5E). To explain how the complex of AFF4/PAX2 regulates *HPRT1* and *IMPDH2* expression, we performed a Ch-IP assay to measure whether AFF4 is required for initiating *HPRT1* and *IMPDH2* gene expression using antibodies against CBP, H3K9ac and RNA pol Ⅱ. As shown in Figure 5F, loss of AFF4 impaired the activated state of *HPRT1* and *IMPDH2* genes on the promoters (Figure 5F and S4A). To identify the correlation between AFF4 and PAX2, we stably depleted PAX2 in BxPC-3 and AsPC-1 cells (Figure S4B). PAX2 depletion reduced the binding of AFF4 to the promoter of *HPRT1* and *IMPDH2* genes, further proving PAX2 is required for recruiting AFF4 on the promoter of *HPRT1* and *IMPDH2* in BxPC-3 and AsPC-1 cells (Figure 5G and S4C). Together, AFF4 drives HPRT1 and IMPDH2 expression depending on transcriptional factor PAX2.

### Phosphorylation of AFF4 at S388 determined the recruitment of AFF4 for PAX2

**Figure 6:**
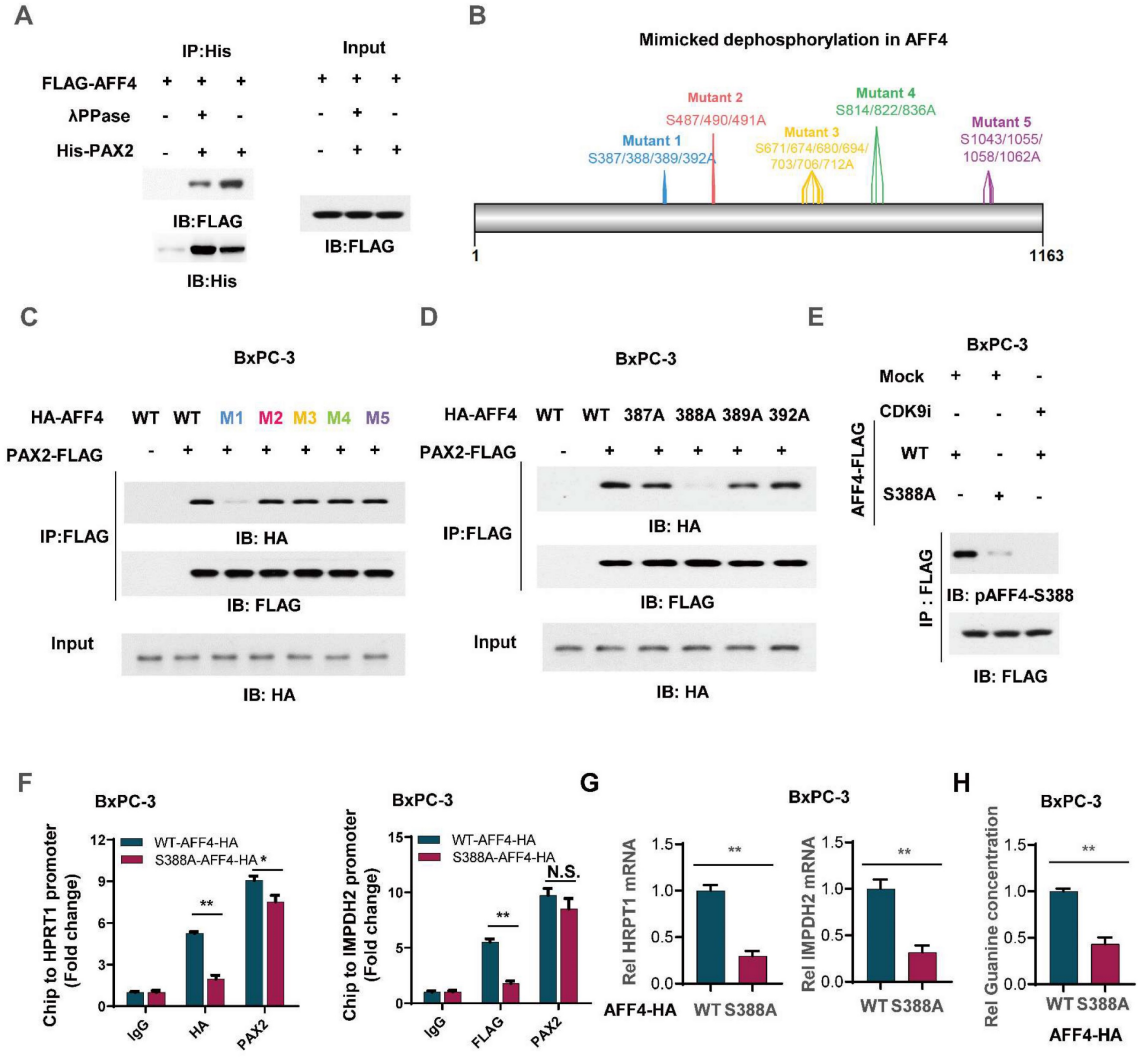
**Phosphorylation of AFF4 at S388 determined the recruitment of AFF4 for PAX2.** (A) FLAG-AFF4 was enriched from BxPC-3 cells and washed with 0.5% NP40, treated with or without λPPase (10 nM) for one hour, then incubated with His tagged PAX2 which was purified from *E.coli*. (B) The schematic diagram of mimicked dephosphorylation mutation strategy in AFF4. (C) HA-tagged AFF4, including WT and five serine cluster mutants, were transfected with or without FLAG-tagged PAX2 into BxPC-3 cells, and FLAG-M2 beads were used to immuno-precipitate FLAG-tagged PAX2 for Co-IP assay. (D) HA-tagged AFF4, including WT and distinct serine mutants, were transfected with or without FLAG-tagged PAX2 into BxPC-3 cells, and FLAG-M2 beads were used to immuno-precipitate FLAG-tagged PAX2 for Co-IP assay. (E) BxPC-3 cells stably expressing WT or S388A mutant were treated with or without CDK9 inhibitor, JSH-150,100nM for 6 hours. (F-H) ChIP assay was performed using antibodies against HA and PAX2 (F), qPCR was performed using specific primers against *HPRT1* mRNA(G), and relative intracellular concentration of guanine is determined by using LC-MS (H) in BxPC-3 cells stably expressing WT or S388A mutant. Data in triplicate from (F-H). F-H, two-tailed Student's t-test. (n.s., not significant; *p < 0.05; **p < 0.01).

To clarify the mechanism that how PAX2 recruits AFF4, we first applied λPPase to treat flag-tagged AFF4 which was derived from BxPC-3 cells and then incubate it with purified PAX2 from *E. coli*. As shown in Figure 6A, the pretreatment of λPPase for AFF4 directed the lost association of AFF4 with PAX2, indicating the phosphorylation in AFF4 would contribute to the formation of AFF4/PAX2 axis (Figure 6A). As phosphositeplus (www.phosphosite.org) showed that there are five phosphorylation clusters in AFF4 and we mutated these serine sites to alanine sites (Figure 6B). Co-IP assay tested the association of AFF4, including wild-type and five mutants, and data showed that the deficiency of phosphorylation in the cluster one: S387/388/389/392, would dramatically impair the formation of AFF4/PAX2 axis (Figure 6C). We further ascertained that the phosphorylation of AFF4 at S388 determined the recruitment of AFF4 for PAX2 (Figure 6D). As we observed that in the AFF4 associated complex, CDK9 is the prominent kinase which is required for serine phosphorylation, we suspected CDK9 could be tightly correlated with AFF4 phosphorylation at S388. For this, we prepared antibody specially recognized AFF4-S388 phosphorylation and tested the specificity of this antibody by dot blot (Figure S5A), then used CDK9 inhibitor JSH-150 pretreated BxPC-3 cells (Figure 6E) or siRNA targeting CDK9 coding gene (Figure S5B) and found a significant decrease of AFF4-S388 phosphorylation upon CDK9 inhibition or depletion (Figure 6E and Figure S5B). We also confirmed whether the disruption of AFF4/PAX2 would affect AFF4 targeting the promoters of *HRPT1* and *IMPDH2*. As shown in Figure 6F, the loss of AFF4-S388 phosphorylation induced a heavy decrease of AFF4 accumulation on the promoters of *HRPT1* and *IMPDH2*(Figure 6F). Consistently, the expression levels of *HRPT1* and *IMPDH2* were also reduced (Figure 6G) and the levels of intracellular guanine were decreased as expected (Figure 6H). Taken together, in the AFF4/PAX2 axis, CDK9 phosphorylated AFF4 at S388 and tightened this axis for activating targeted gene expression.

### PI3K/c-Myc axis promotes AFF4 expression in pancreatic cancer cells

**Figure 7:**
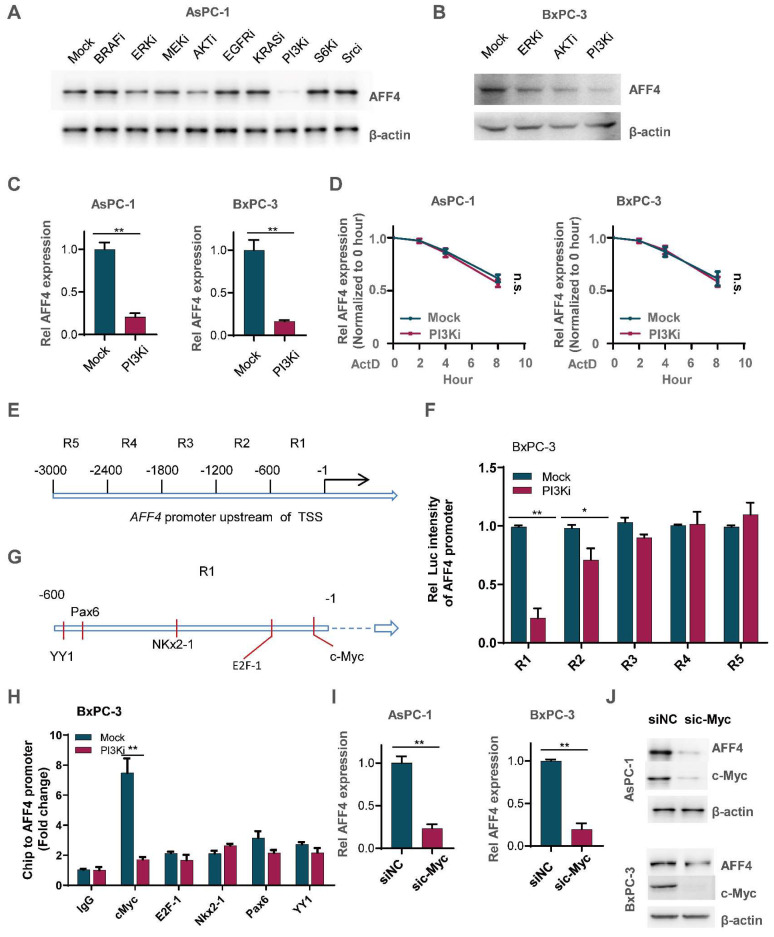
** PI3K/c-Myc axis promotes AFF4 expression in pancreatic cancer cells.** (A) Inhibitors screen was exerted on BxPC-3 cells and western blot analysis of AFF4 expression was performed. KRASi, 5 μM; BRAFi, 2 μM; AKTi, 1 μM; EGFRi, 1 μM; MEKi, 2 μM; ERKi, 0.5 μM; PI3Ki, 1 μM; S6Ki, 0.2 μM; Srci, 2 μM. All of inhibitors were incubated with cells for 12 hrs. (B) Inhibitors of ERK, AKT or PI3K were used to treat BxPC-3 cells for 12 hrs. (C) qRT-PCR analysis of AFF4 expression in BxPC-3 or AsPC-1 cells with or without PI3K inhibitor for 12 hrs. (D) mRNA decay assay. AsPC-1 or BxPC-3 cells were incubated with ActD and PI3K inhibitor under the treatment of ActD for indicated times. ActD, 1μM. (E) Schematic division of the 3000 bp upstream of the AFF4 transcriptional start site (TSS). (F) R1 and R2 were required for stimulating AFF4 mRNA expression. R1, region 1; R2, region 2. Dual luciferase reporter assay was used to measure AFF4 promoter activity. (G) The predicted transcription factors by PROMO binding on the AFF4 promoter R1. (H) ChIP assays were performed to find the transcription factors required for binding of the AFF4 R1. (I-J) AFF4 mRNA (I) or protein level (J) was downregulated accompanied by knocking down of c-Myc in AsPC-1 and BxPC-3 cells. Data in triplicate from (C and F-H). C and F-H, two-tailed Student's t-test. D, two-way ANOVA. (n.s., not significant; *p < 0.05; **p < 0.01).

To ascertain how AFF4 expression is regulated in PDAC, we tested the signaling pathway required for maintaining the protein level of AFF4 using specific inhibitors. As shown in Figure 7A, inhibition of PI3K by LY294002 heavily reduced AFF4 protein level. Meanwhile, inhibition of ERK by LY3214996 or inhibition of AKT by MK-2206 moderately reduced AFF4 protein level. Of note, PI3K was known to function in the upstream of AKT in multiple types of cancers, especially ERK and AKT (Figure 7A-B). And the reduced protein level could be due to the decreased *AFF4* mRNA level by inhibition of PI3K activity (Figure 7C). We also tested the mRNA decay using Actinomycin D and we observed the same trend of mRNA decay half-time, meaning PI3K signal mediated *AFF4* mRNA downregulation is regulated in the transcriptional level, but not mRNA stability (Figure 7D).

How does PI3K signals for initiating *AFF4* expression? As AFF4 is regulated by PI3K in the transcriptional level, we first divided the 3000 base pairs (bp) upstream of the TSS in *AFF4* gene into 5 regions (Figure 7E). These sequences were separately cloned into the pGL3 vector and then transfected into BxPC-3 cells which were treated with or without PI3K inhibitor. A dual luciferase report assay showed us that region 1 and 2 (R1 and R2), particularly R1 in the PI3K inhibitor treated cells, were required for activating AFF4 transcription (Figure 7F). Predictions using the website PROMO showed that five transcriptional factors-c-Myc, E2F1, Nkx2-1, Pax6 and YY1-may potentially bind R1 region (Figure 7G). A Ch-IP assay showed that the targeting of AFF4-R1 by c-Myc significantly decreased under treatment of PI3K inhibitor in BxPC-3 cells (Figure 7H). PI3K inhibition induced instability of c-Myc reduced AFF4 levels in both levels of protein and mRNA, further proving c-Myc regulates AFF4 in mRNA expression (Figure S6A-C). Meanwhile, we excluded the possibility that c-Myc directly targeted the promoters of *HPRT1* and *IMPDH2* in PDAC cells, further insisted the idea that c-Myc mediated AFF4 expression and then indirectly upregulated nucleotide metabolism (Figure S6D and S6E). Otherwise, we further identified c-Myc is required for AFF4 mRNA and protein expression in AsPC-1 and BxPC-3 cells (Figure 7I and 7J). Together, data above preliminarily showed PI3K/c-Myc axis was required for maintaining AFF4 expression in PDAC cells.

### c-Myc, AFF4 and its downstream targets HPRT1 and IMPDH2 implicate a clinical significance

**Figure 8:**
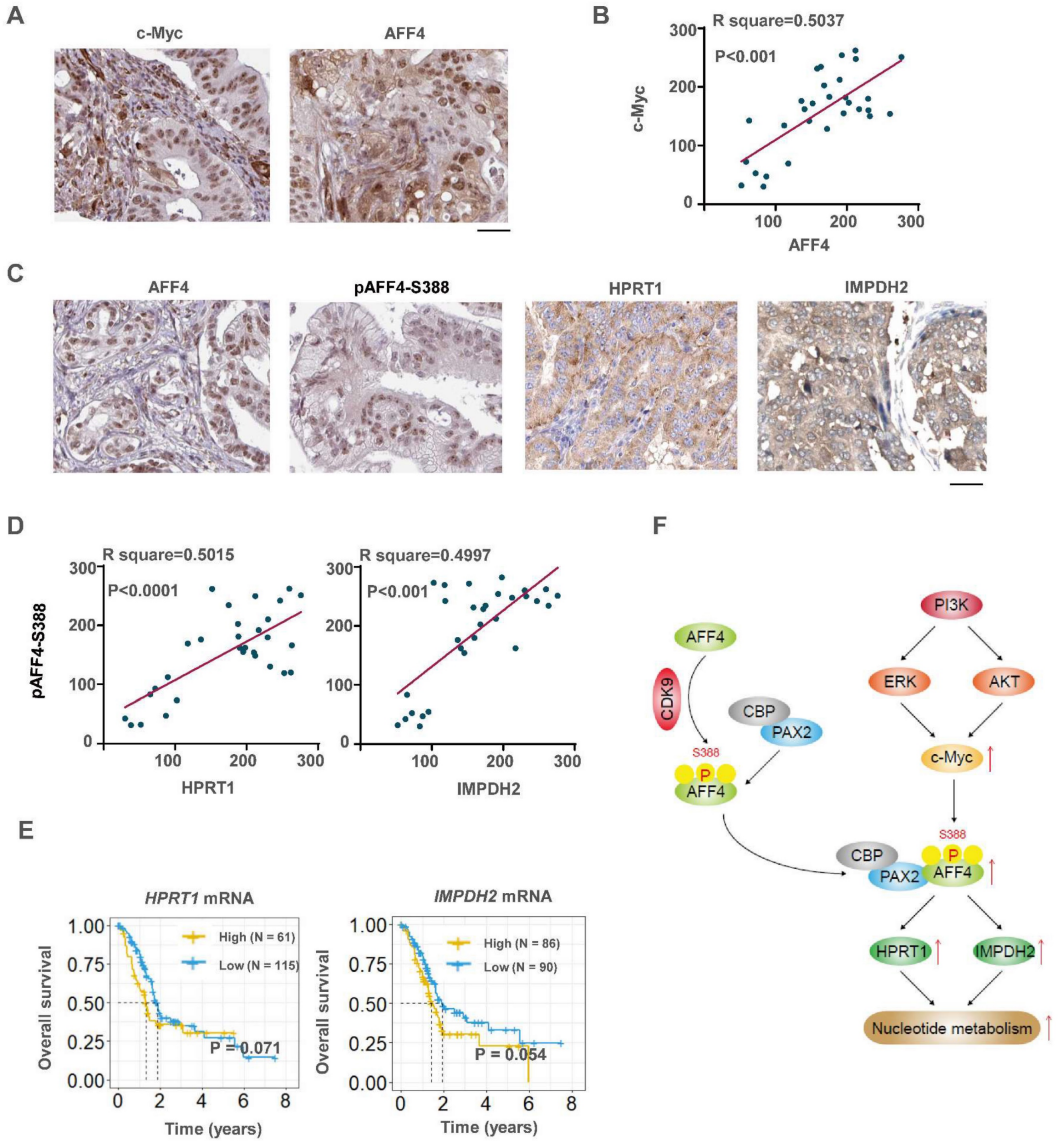
** c-Myc/AFF4 axis and its downstream targets HPRT 1and IMPDH2 implicate a clinical significance.** (A-B) IHCs of PDAC clinical samples were performed using antibodies against c-Myc and AFF4, respectively (A). The correlated IHC signal between c-Myc and AFF4 (B) in PDAC samples was calculated. (C-D) IHCs of PDAC clinical samples were performed using antibodies against AFF4, HPRT1 or IMPDH2 (C). The correlated IHC signal between AFF4 and HPRT1 (D, left panel) or AFF4 and IMPDH2 (D, right panel) in PDAC samples were calculated. (E) Overall survival of patients in the TCGA-PDAC cohort (Split patients by: best separation; Follow up threshold: 80 months). (F) The mechanism in this study. (A) and (C), Scale bar, 200 μm; (B) and (D), two-tailed Student's t-test.

To investigate the clinical significance of c-Myc and its downstream AFF4, or AFF4 and its downstream targets HPRT1 and IMPDH2, IHC analyses were performed with 30 specimens from human primary PDAC patients using indicated antibodies (Figure 8A). As shown in Figure 8B, c-Myc levels were positively correlated with the levels of AFF4. Pearson correlation analysis of the staining on a scale of 0-300 showed that these correlations were significant (Figure 8B).

Then we performed IHC with above specimens using anti-AFF4, anti-HPRT1 or anti-IMPDH2 antibodies (Figure S7A and 8C). As shown in Figure 8D, AFF4 levels were positively correlated with levels of HPRT1 and IMPDH2 with a similar extent. Pearson correlation analysis of the staining on a scale of 0-300 showed that these correlations were significant (Figure 8D), suggesting the existence of a clinically relevant AFF4/ HPRT1&IMPDH2 axis. Further, the survival curves of AFF4 were also plotted by mining TCGA-PDAC database and showed that higher levels of AFF4 mRNA predicts worse prognosis in PDAC patients. And the overall survival curves of HPRT1 and IMPDH2 also indicated that high expression of HPRT1 and IMPDH2 conveys worse prognosis, with the same trend as AFF4, although the difference is not significant in TCGA-database (Figure 8E). Together, clinical data suggests a higher expression of AFF4 driving the expression of HPRT1 and IMPDH2 constrains PDAC patients' survival duration.

## Discussion

Super elongation complex (SEC) is essential for gene expression and the dysregulation of SEC is regarded as the original reason of diseases. To clarify the role of AFF4, a subunit of SEC, in pancreatic tumorigenesis, we first performed functional experiments, such as cell proliferation, colony formation, cell cycle and xenograft studies to confirm the oncogenic role of AFF4 in PDAC cells. Then, we explored the mechanism that AFF4 associates with PAX2 to drive *HPRT1* and *IMPDH2* expression and in turn promotes cell cycle progression in PDAC cells by fueling nucleotide metabolism. In detail, CDK9 mediated phosphorylation of AFF4 at S388 promotes AFF4/PAX2 to form an axis which drives downstream gene expression by remodeling the state of gene promoters. In the upstream of AFF4, we explored that PI3K signals for AKT and ERK to activate AFF4 expression. Thus, our study explores the relationship between AFF4 and nucleotide metabolism, revealing an oncogenic role for AFF4 in PDAC, which may represent a potential target for PDAC treatment (Figure 8F).

AFF4 showed prompting functions in multiple cancer types, such as head and neck squamous cancer 6, melanoma 7, two kinds of glioma (diffuse intrinsic pontine glioma and diffuse midline glioma) 9, 14 and bladder cancer 15, 16. In this study, a series of functional experiments established the oncogenic role of AFF4 in pancreatic cancer. However, how AFF4 promotes pancreatic tumorigenesis remains elusive. We applied RNA sequencing to discover the differential genes regulated by AFF4 and revealed that 208 genes were upregulated and 143 genes were downregulated accompanying AFF4 overexpression. Among the 208 upregulated genes by AFF4 overexpression, there were two genes, *HPRT1* and *IMPDH2*, which encode rate-limiting enzymes in the nucleotide metabolic pathway.

In BioGrid database (https://thebiogrid.org/), AFF4 has 63 interactors derived from 131 ineractions, including elongation factors, such as ELL2/ELL/ELL3, ENL/AF9; and histones, such as H2AFZ, HIST1H1B, HIST1H2BH and HIST1H3A; Cell dependent kinases, such as CDK9/8/15; Cyclin T1/T2; Epigenetic modification enzymes and mediators, such as KMT2A, MED26/MED19. In this study, we performed a LC/MS-MS to identify the AFF4 interactors in the PDAC cells and we observed a transcriptional factor, PAX2 which targets special DNA elements on the promoter or enhancer for gene expression. Of note, we also observed MLLT1/3, CDK9 which were shown as the interactors of AFF4 in the BioGrid database. Hence, our mass identification result is mainly consistent with previous study. Also, we identified that CDK9 mediated phosphorylation of AFF4 especially at S388, which in turn reinforces the axis of AFF4/PAX2 and facilitates upregulating downstream gene expression by remodeling the chromatin state on the promoters of genes.

Growing evidence supports that activation of several oncogenes such as KRAS and c-Myc can induce metabolic reprogramming and oncogenic KRAS is the key driver of PDAC 17, 18. The KRAS-driven metabolic changes were mediated by the RAF/MEK/ERK pathway, which results in upregulation of c-Myc and the transcriptional regulation of rate-limiting enzymes in glucose metabolism 19. Also, the dependence on oncogenic KRAS correlates with specific metabolic profiles that involve maintenance of nucleotide pools as key mediators of KRAS-dependence 10. Therefore, it is necessary to study whether KRAS drives AFF4 to up-regulate the genes expression of *HPRT* and *IMPDH2* through KRAS/MEK/ERK signaling pathway. However, in this study, we observed that inhibition of PI3K, but not KRAS, heavily impaired AFF4 expression. PI3K dysregulation occurs in a wide spectrum of human cancers and considerable efforts have been focused on the development of PI3K inhibitors with a view to clinical application. However, to date the clinical outcome of PI3K inhibitor-based treatments for solid tumors has been disappointing, which could be due to many reasons. And serious side effects from inhibitor toxicity are another adverse factor for application of PI3K inhibitor 13, 20. To clarify the regulatory circuit between PI3K/c-Myc and AFF4, we forced expression of wild-type or T58A/S62A mutant c-Myc with or without PI3K inhibition. As shown, PI3K inhibition heavily blocked the c-Myc protein levels, but not c-Myc mRNA levels (Figure S6A-B). In detail, we used cMyc-T58A/S62A mutant which was more stabilized in the protein level. Upon PI3K inhibition, the abundance of cMyc-WT protein, but not cMyc-T58A/S62A mutant, was reduced, suggesting PI3K regulates c-Myc in the level of protein stability (Figure S6A). and qRT-PCR proved this speculation (Figure S6B). Of note, inhibition of ERK activity also reduced AFF4 expression and we speculate that ERK crossed with PI3K signaling pathway and was responsible for transmitting part of PI3K signal to downstream.

c-Myc directly regulates about 1,200 target genes that affect many aspects of tumor behavior, such as proliferation, growth, metastasis, metabolic abnormality and drug resistance 21. In this study, we identified a novel direct target of c-Myc, AFF4, which in turn simultaneously upregulates HRPT1 and IMPDH2 expression. However, Ch-IP assay showed that c-Myc didn't directly target *HRPT1*'s and *IMPDH2*'s promoters. Thus, c-Myc also indirectly fuels nucleotide metabolism by the c-Myc/AFF4 axis as c-Myc has been reported directly target genes coding enzymes in the glucose 22, 23, pentose 24, 25 or glutamine metabolic pathways 26, 27.

We further examined AFF4, HPRT1 and IMPDH2 expression in clinical samples and revealed the clinical significance of AFF4, HPRT1 and IMPDH2. We observed a positive correlation between the expression levels of AFF4 and HPRT1 or IMPDH2, suggesting the axis of AFF4/HPRT1 or IMPDH2 would also exist in the clinical samples. Although, the survival curves of HPRT1 and IMPDH2 had a p value near 0.05, these curves showed a trend that high expression predicted a worse prognosis of PDAC patients.

## Conclusion

Conclusively, AFF4 is not only a biomarker of PDAC, but also plays as an oncogenic role in PDAC progression, hence, offers a therapeutic target for PDAC.

## Supplementary Material

Supplementary materials and methods, figures and table.
