# Supplementary material for: PI3K/ c-Myc/AFF4 axis promotes pancreatic tumorigenesis through fueling nucleotide metabolism
Source: Int J Biol Sci. 2023 Mar 27;19(6):1968–82. doi: 10.7150/ijbs.77150 (PMC10092763; doi:10.7150/ijbs.77150)
Supplement: Supplementary file 1 — Supplementary materials and methods, figures and table. [file ijbsv19p1968s1.pdf]

Supplementary Materials and Methods

Antibodies

Rabbit polyclonal or monoclonal antibodies against AFF4 (PA5-103584, 1:200 for IHC, 1:1000 for IB), HPRT1 (MA5-15274, 1:200 for IHC, 1:1000 for IB), IMPDH2 (PA5-27494, 1:200 for IHC, 1:1000 for IB) and c-Myc (MA1-980, 1:200 for IHC, 1:2000 for IB) were purchased from Thermo Fisher (Lafayette, CO, USA); Mouse monoclonal anti-actin (MA1-744, 1:5000 for IB) and anti-Ki67 (SAB5300423, 1:500 for IHC) were purchased from Sigma (Lafayette, CO, USA).

Rabbit polyclonal or monoclonal antibodies against c-Myc (ab56, 1:60 for ChIP), E2F1 (ab179445, 1:60 for ChIP), Nkx2-1 (MA5-13961, 1:60 for ChIP), Pax6 (#60433, 1:60 for ChIP), CDK9 (MA5-14912,1:1000 for WB), HA(26183, 1:5000 for WB, 1:100 for ChIP or IP), FLAG(740001, 1:5000 for WB, 1:100 for IP) and YY1 (MA5-15274, 1:200 for IHC, 1:1000 for IB) were purchased from Abcam (Suzhou, China), Thermo Fisher (Lafayette, CO, USA) or Cell signaling Technology (Beverly, CA, USA). Antibody against AFF4-S388 phosphorylation was designed and made by Abclonal (Wuhan, China).

Reagents

KRASi, AMG510 (HY-114277); BRAFi, GDC-0879 (S1104); AKTi, MK-2206 2HCl (S1078) ; EGFRi, Afatinib (BIBW2992, S1011); MEKi, PD0325901 (S1036); ERKi, LY3214996 (S8534); PI3Ki, LY291002 (S1105); S6Ki, LY2584702 (S7698); Srci, Src Inhibitor-1(S2075); Cycloheximide (CHX, S7418); Actinomycin D (S8964) and CDK9 inhibitor, JSH-150 (S8783) were purchased from Sigma-Aldrich (Lafayette, CO, USA) or Selleck (Houston, TX, USA). λPPase (p0753) was purchased from NEB.

c-Myc siRNA was purchased from Cell signaling technology (#6341). CDK9 siRNA was purchased from Thermofisher (AM16708).

Cell culture and transfection

AsPC-1, BxPC-3, CFPAC-1, CAPAN-2, MIAPaCa2, PANC-1 and SW1990 were purchased from American Type Culture Collection (ATCC). PaTu8988 was a gift from Dr. Weiwei Yang (CAS, China). Cell lines were tested for c-Mycoplasma contamination before use to ensure that they were mycoplasma-free.

Plasmids were transfected into PDAC cells using the Lipo3000^{TM} transfection reagent (Life Technologies, USA) while a non-specific plasmid (shNT or Vec) was used as a negative control.

Lentivirus package and infection

Amplification of lentivirus was via standard methods in sub confluent HEK293T cells. Infection of pancreatic cancer cell lines was performed in the presence of polybrene (Sigma) at a final concentration of 8 μg/ml. Cells were incubated with lentivirus mixture for 72 hours and digested with trypsin to fresh growth medium, then sorted with green fluorescence for stable expression or knockdown. The constructed stable cell lines were amplified and saved for future experiments.

Cell proliferation assay

Pancreatic cancer cells with or without genetic modifications were plated in DMEM (10% FBS) or RIPM1640 (10% FBS). Cells were collected in the indicated time point and cell proliferation rate was determined by CCK8 kit (Dojindo Molecular Technologies, Japan).

Colony formation assay

For colony formation assays, 5000 cells per well were plated in six-well plate in triplicates and cultured for 14 days before staining viable colonies with nitro blue tetrazolium (Sigma).

FACS analysis of cell cycle

The indicated cells in this study were starved by serum-free medium for 16 or 24 hours to be synchronized at G0 phase. After that, the cells were then released to fresh medium supplemented with 10% serum for additional time (from zero to 16 or 24 hours) and collected at the indicated time points. After a phosphate-buffered saline (PBS) wash, the cells were fixed in ice-cold 70% EtOH for 2 hours at 4°C and centrifuged at 2,000 rpm for 5 min, followed by two PBS washes. The cells were then incubated in 37°C RNase solution (1 mg/ml) for 30 min and stained with propidium iodide (50 μg/ml) for cell cycle analysis (488 nm excitation). A Galios Flow Cytometer (Beckman) was used for pulse processing and collecting fluorescence above 620 nm emission, and data were processed with Modfit software (Verity Software House, ME, USA).

RNA profiling and Quantitative RT-PCR (qRT-PCR)

Total RNA was extracted with an RNA high-purity total RNA rapid extraction kit (QIAGEN, 74034) and RNA profiling and data collection, analyzation were performed in Decode Gene (Shanghai). cDNA was prepared using a GoScript reverse transcription system (Promega, TM316). qRT-PCR was performed using SYBR PCR premixture (Promega, M7502) under the following conditions: 5 min at 95^0C followed by 38 cycles at 95^0C for 30 s, 60^0C for 40 s, and 72^0C for 1 min using an ABI

7500 fast system. Data were normalized to the expression of the control gene (β-actin) for each experiment. Data represent the mean ± SD of three independent experiments. The sequences of primer pairs used for qRT-PCR were listed in the Supplementary Table 1.

Luciferase reporter gene assay

The transcriptional activation of different region in the AFF4 promoter in BxPC-3 cells was measured using a Dual-Luciferase Assay Kit (Promega, E1960) on a GloMax 20/20 luminometer (Promega, E1910) following the manufacturer's instructions. In detail, the cells were plated in triplicate at a density of 2×10^4 cells/well in a volume of 500 μl in separate 24-well microtiter plates. After transfection with the indicated plasmids for 48 hours, the cells were washed with cold PBS and lysed with lysis buffer. The relative levels of luciferase activity were normalized to the levels in untreated cells and to the levels of Renilla luciferase activity of the control plasmid in each group. Primers for constructing AFF4 promoter are listed in Supplemental Table 1.

Antibody generation

Antibodies against AFF4 phosphorylation at S388 were custom designed and constructed by ABclonal Technology (Wuhan, China). Briefly, the phospho-peptides pho-S388-AFF4 and their respective control peptides without phosphate group were synthesized. New Zealand White rabbits are immunized by intradermal injection of 500 μg of indicated polypeptides which were mixed with Freunds adjuvant to 2ml volume. Repeating this process three times, reducing the dose to half in the fourth time. Serum samples were taken to test the titers of indicated antibodies at 2 weeks after the fourth immunization. The antibody is purified and obtained by a repeat of affinity chromatography.

Chromatin immune-precipitation (ChIP) assay

For ChIP experiments, the indicated cells were fixed in 1% formaldehyde for 10 min for pull-down of c-Myc, E2F1, NKX2-1, Pax6 and YY1. The cells were rotated in cold lysis buffer (10 mM Tris-HCl pH 7.4, 10 mM NaCl, 3 mM MgCl2, 0.5% NP40, supplemented with freshly prepared PMSF and protease inhibitor cocktail) for 30 min. Then, the nuclear pellets were resuspended in RIPA buffer (300 mM NaCl, 3 mM EDTA, 1% NP40, 0.5% sodium deoxycholate, 0.1% SDS, 100 μg/ml BSA, 50 mM Tris-HCl pH 7.5) and sonicated with a Covaris S220 to yield DNA fragments of approximately 200–500 bp. A ChIP-grade antibody was incubated with 30 μl of protein G Dynabeads (Thermo Fisher, 10003D) for 4 hours. Then, the DNA fragments were coimmunoprecipitated with the specific antibody-conjugated protein G beads at 4°C overnight. The DNA was purified with a MinElute PCR Purification Kit (QIAGEN, 28004). The promoter regions

of AFF4 were amplified and quantified by qRT-PCR using SYBR Green (Takara, RR820A) on an ABI 7500 Fast system. For PCR, 0.02 ng of the immunoprecipitated DNA and 2 ng of the total DNA were used in a 20 μl reaction. The results from each immunoprecipitation were normalized to the respective inputs. The primers are listed in Supplemental Table 1.

Supplementary Figure and Legends

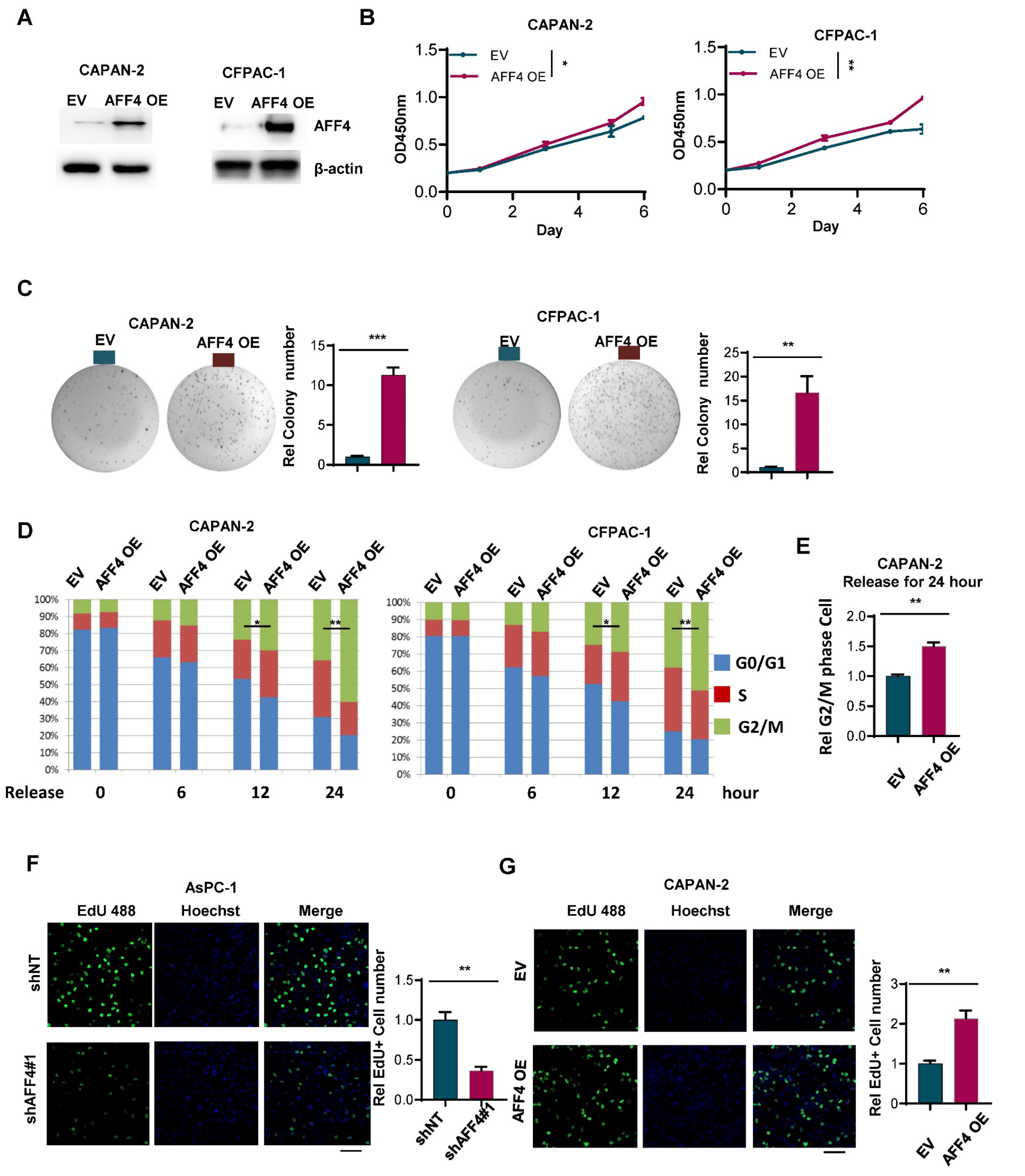

Figure S1. AFF4 overexpression promotes pancreatic cancer cell proliferation, colony formation and fuels cell cycle progression. (A) Western blot confirmed the successful construction of cell lines stably expressing AFF4. (B-D) Cell proliferation assay (B), colony formation assay (C) and cell cycle assay (D) were performed in CAPAN-2 or CFPAC-1 cells with EV or AFF4 OE. (E) The indicated cells were released for 24 hrs from cell cycle arrest and collected for PI staining and cell cycle analysis. (F-G) EdU staining was performed in AsPC-1 cells with or without AFF4 (F), or in CAPAN-2 cells with or without AFF4 overexpression (G). EV, empty vector; OE, overexpression. Data in triplicate from (C-G). B, two-way ANOVA. C-G, two tailed Student's t-test.

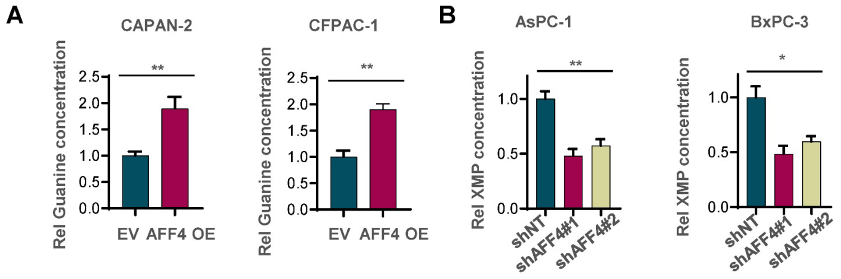

Figure S2. (A) In CAPAN-2 and CFPAC-1 cells with or without AFF4 overexpression, relative concentration of guanine is determined using LC-MS. (B) In AsPC-1 and BxPC-3 cells with or without AFF4, relative concentration of XMP is determined using LC-MS. Data in triplicate from (A-B). A-B, two tailed Student's t-test.

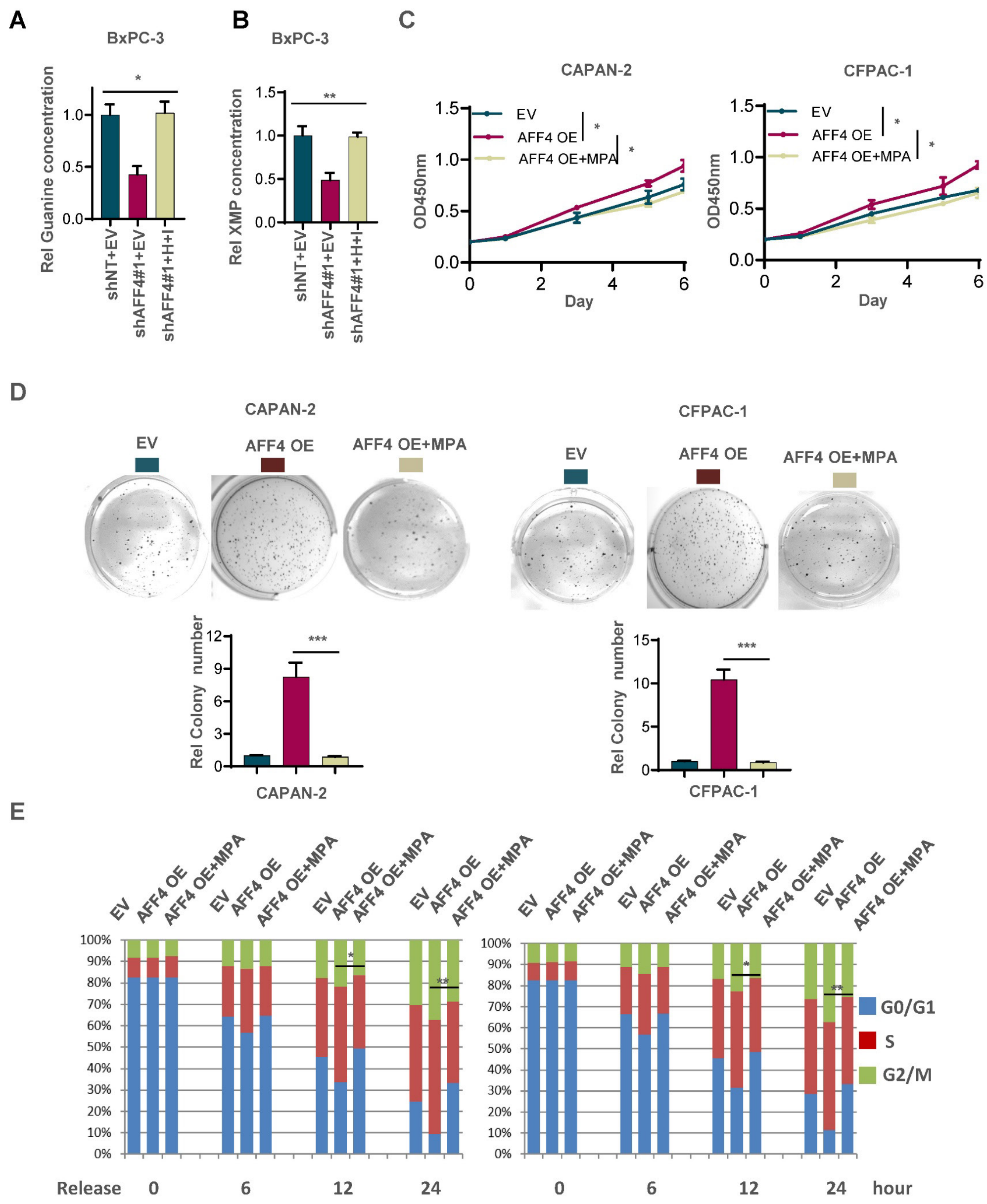

Figure S3. Inhibition of IMPDH2 impaired the prompted function of AFF4 in pancreatic cancer cells. (A-B) In BxPC-3 cells with or without AFF4, relative concentration of guanine (D) or XMP (E) is determined using LC-MS. (C-E) Cell proliferation assay (C), colony formation assay (D) and cell cycle assay (E) were performed in CAPAN-2 or CFPAC-1 expressing AFF4 OE with or without MPA treatment. EV, empty vector as a negative control; OE, overexpression; MPA, Mycophenolic acid. Data in triplicate from (A-B). A, two-way ANOVA. A-B and D-E, two tailed Student's t-test.

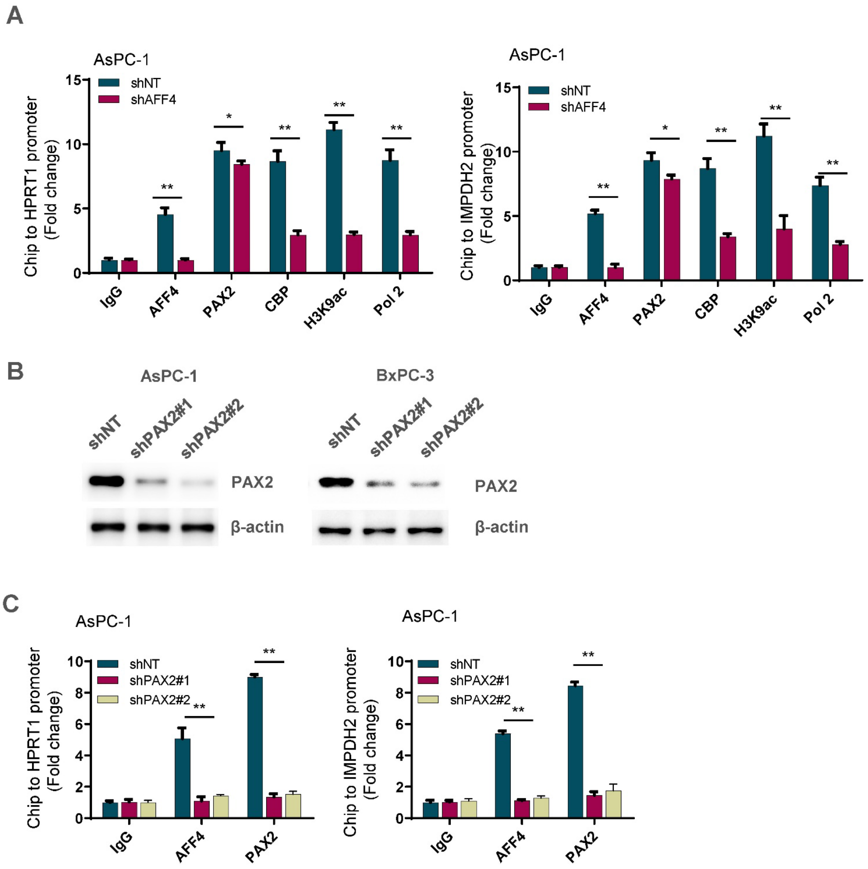

Figure S4. PAX2 specifically recruited AFF4 on the promoter of *HPRT1* and *IMPDH2* genes. (A) ChIP assay was performed using antibodies against AFF4, PAX2, CBP, H3K9ac and RNA pol II in AsPC-1 cells expressing shNT or shAFF4. (B) Western blot confirmed the successful construction of AsPC-1 or BxPC-3 cell lines stably depleting PAX2. (C) ChIP assay was performed using antibodies against AFF4 and PAX2 in AsPC-1 cells expressing shNT or shPAX2. A and C, two-tailed Student's t-test.

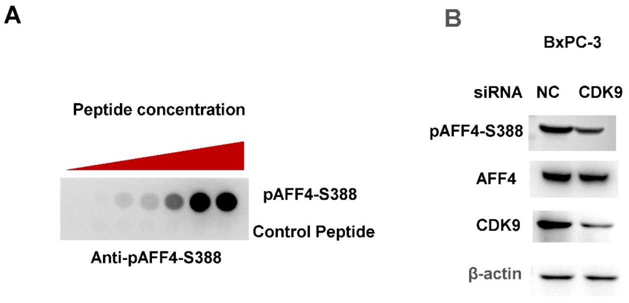

Figure S5. (A) pAFF4-S388 peptide and control peptide without AFF4-S388 phosphorylation were loaded and blotted with indicated antibody. (B) siRNA targeting CDK9 or non-target control were transfected into BxPC-3 cells for 56 hours. WB was performed using indicated antibodies.

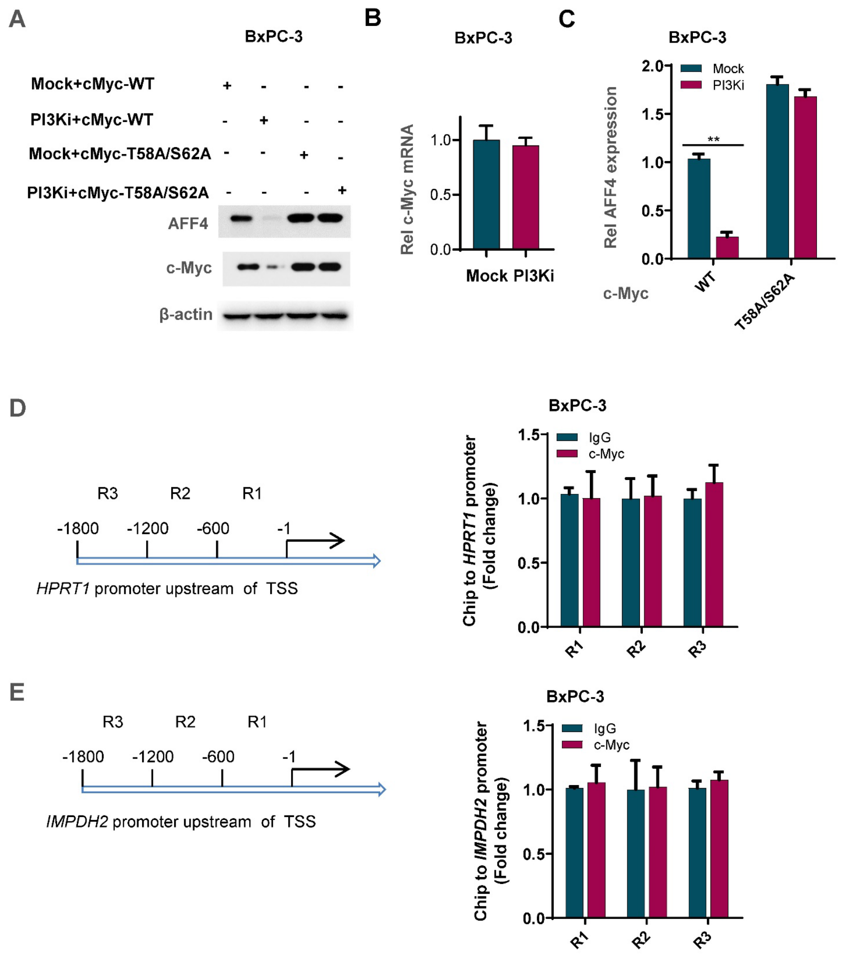

Figure S6. c-Myc targeted *AFF4* promoter but didn't target the promoter of *HPRT1* and *IMPDH2* genes. (A-C) In BxPC-3 cells, WT or T58A/S62A mutant c-Myc were overexpressed, then treated with or without PI3K inhibitor (1 μM) for 6 hours. WB using indicated antibodies (A) or qRT-PCR (B-C) using specific primers against c-Myc or AFF4 mRNA was performed. (D) Schematic division of the 1800 bp upstream of the *HPRT1* gene TSS(Left). ChIP assays using antibody against c-Myc were performed (Right). (E) Schematic division of the 1800 bp upstream of the *IMPDH2* gene TSS(Left). ChIP assays using antibody against c-Myc were performed (Right).

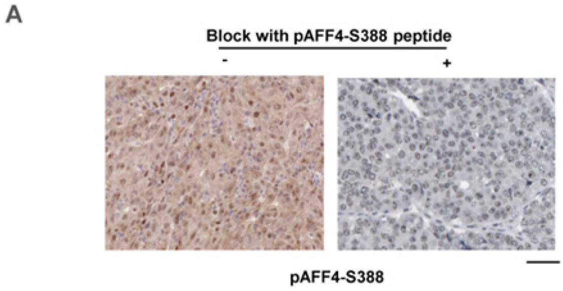

Figure S7. The specificity of pAFF4-S388 antibody was ascertained using peptide blocking assay.

Supplementary Table 1 (primers used in this study)

Primers for constructs

UNI-CMV-AFF4-F
GTGGAATTCGCTAGCGGATCC atgaaccgtgaagaccggaat
UNI-CMV-AFF4-R
ATGTCGACCTCGAGTGCGGCCGC tcaagatatcaacttggcatc

UNI-pGL3B-AFF4(-3000～-2400)-F
GTGGAATTCGCTAGCGGATCC TTGCTCTTGTCACCCAGGCT
UNI-pGL3B-AFF4(-3000～-2400) -R
ATGTCGACCTCGAGTGCGGCCGCATCCTGAAAGATCTGCAACTT

UNI-pGL3B-AFF4(-2400～-1800)-F
GTGGAATTCGCTAGCGGATCC TTAAATTTAAGGAAACTGGT
UNI-pGL3B-AFF4(-2400～-1800) -R
ATGTCGACCTCGAGTGCGGCCGCAAAAAGGGACCCGAGGTGGG

UNI-pGL3B-AFF4(-1800～-1200)-F
GTGGAATTCGCTAGCGGATCCTTTTCTAAACATTAAATTGCCT
UNI-pGL3B-AFF4(-1800～-1200) -R
ATGTCGACCTCGAGTGCGGCCGCCTGAGTAGCTGGGATTACAG

UNI-pGL3B-AFF4(-1200～-600)-F
GTGGAATTCGCTAGCGGATCCGAGGCTGAGGTAGGAGAATC
UNI-pGL3B-AFF4(-1200～-600) -R
ATGTCGACCTCGAGTGCGGCCGCCTCAATAAATGGCAAAACCC

UNI-pGL3B-AFF4(-600～-1)-F
GTGGAATTCGCTAGCGGATCCGAGGTGTTAATTGAAATATTA
UNI-pGL3B-AFF4(-600～-1) -R
ATGTCGACCTCGAGTGCGGCCGCGTTGCTATGAAAAGAAACAC

Sequences of Primers for qRT-PCR

Homo-AFF4-F 5'- TCTCAGTCTCAGAAACGGTCC
Homo-AFF4-R 5'- GGCTACTGCTCCCACTATTGTT

Homo-HRPT-F 5'-ACCAGTCAACAGGGGACATAA
Homo-HRPT-R 5'-CTTCGTGGGGTCCTTTTCACC

Homo-IMPDH2-F 5'-GCGCTTACAGGCGGTATTG
Homo-IMPDH2-R 5'- AAAACATCCCGCACGCGAT

Homo-c-Myc-F 5'- CGATGTGGGAACTGGGTACTA
Homo-c-Myc-R 5'- ATCATTTCCATGACGGCCTGT

Sequences of Primers for ChIP-qPCR
Homo-AFF4-F: GTCAGTCACAGCCCTGGCTGC
Homo-AFF4-R: TCTGCAAAGAGAGGAGAGCTA

Homo-HPRT1-F(R1): GATAATTTTTCATTTTTGTAG
Homo-HPRT1-R(R1): GGGAGGCGGAGGTCGCACTGA

Homo-HPRT1-F(R2): TCAGTGCGACCTCCGCCTCCC
Homo-HPRT1-R(R2): CCTCCCAACTCAGTCTCCTA

Homo-HPRT1-F(R3): TAGGAGACTGAGTTGGGAGG
Homo-HPRT1-R(R3): CACCTTCTCTTCCCACACGCA

Homo-IMPDH2-F(R1): GAGAGCCCTAGGCTGGTGCA
Homo- IMPDH2-R(R1): TATTTTAAACAGAAGCAGCC

Homo-IMPDH2-F(R2): GGCTGCTTCTGTTTAAAATA
Homo-IMPDH2-R(R2): TAACATGTTGAAAAGACGTGC

Homo-IMPDH2-F(R3): GCACGTCTTTTCAACATGTTA
Homo-IMPDH2-R(R3): GTGGGTGGCCAATTCCCGAA
